# Genome-wide association study uncovers major genetic loci associated with seed flooding tolerance in soybean

**DOI:** 10.1186/s12870-021-03268-z

**Published:** 2021-10-29

**Authors:** Ripa Akter Sharmin, Benjamin Karikari, Fangguo Chang, G.M. Al Amin, Mashiur Rahman Bhuiyan, Aiman Hina, Wenhuan Lv, Zhang Chunting, Naheeda Begum, Tuanjie Zhao

**Affiliations:** 1 National Center for Soybean Improvement, Key Laboratory of Biology and Genetics and Breeding for Soybean, Ministry of Agriculture, State Key Laboratory of Crop Genetics and Germplasm Enhancement, Nanjing Agricultural University, Nanjing, 210095 China; 2 Jagannath University, Dhaka, 1100 Bangladesh; 3 Department of Crop Science, Faculty of Agriculture, Food and Consumer Sciences, University for Development Studies, Tamale, Ghana

**Keywords:** Soybean, Tolerance to submergence stress, QTL mapping, Candidate genes

## Abstract

**Background:**

Seed flooding stress is one of the threatening environmental stressors that adversely limits soybean at the germination stage across the globe. The knowledge on the genetic basis underlying seed-flooding tolerance is limited. Therefore, we performed a genome-wide association study (GWAS) using 34,718 single nucleotide polymorphism (SNPs) in a panel of 243 worldwide soybean collections to identify genetic loci linked to soybean seed flooding tolerance at the germination stage.

**Results:**

In the present study, GWAS was performed with two contrasting models, Mixed Linear Model (MLM) and Multi-Locus Random-SNP-Effect Mixed Linear Model (mrMLM) to identify significant SNPs associated with electrical conductivity (EC), germination rate (GR), shoot length (ShL), and root length (RL) traits at germination stage in soybean. With MLM, a total of 20, 40, 4, and 9 SNPs associated with EC, GR, ShL and RL, respectively, whereas in the same order mrMLM detected 27, 17, 13, and 18 SNPs. Among these SNPs, two major SNPs, Gm_08_11971416, and Gm_08_46239716 were found to be consistently connected with seed-flooding tolerance related traits, namely EC and GR across two environments. We also detected two SNPs, Gm_05_1000479 and Gm_01_53535790 linked to ShL and RL, respectively. Based on Gene Ontology enrichment analysis, gene functional annotations, and protein-protein interaction network analysis, we predicted eight candidate genes and three hub genes within the regions of the four SNPs with *Cis-*elements in promoter regions which may be involved in seed-flooding tolerance in soybeans and these warrant further screening and functional validation.

**Conclusions:**

Our findings demonstrate that GWAS based on high-density SNP markers is an efficient approach to dissect the genetic basis of complex traits and identify candidate genes in soybean. The trait associated SNPs could be used for genetic improvement in soybean breeding programs. The candidate genes could help researchers better understand the molecular mechanisms underlying seed-flooding stress tolerance in soybean.

**Supplementary Information:**

The online version contains supplementary material available at 10.1186/s12870-021-03268-z.

## Background

Soybean [*Glycine max* (L.) Merr.] is one of the most economically important legume crops, providing protein, oil, carbohydrates, minerals, vitamins, folic acid, fibre, isoflavones and other nutrients for humans and animals [1, 2]. The increased importance of soybeans has resulted in a massive expansion of soybean production in the world. In recent decades, soybean production in the world has increased dramatically from 268.58 million metric tons in 2012/2013 to 360.07 million metric tons in 2018/2019 (Statista 2020; https://www.statista.com). However, sustainable soybean production is threatened by various abiotic stresses, including flooding. The soybean yield is reduced by flooding in the various stages of growth [3–6]. Effect of flooding on soybean includes foliar chlorosis, necrosis, stunted growth, defoliation, reduction in nitrogen fixation and plant death [7–9]. Plants undergo different mechanisms, including morphological, physiological, and biochemical adaptations under flooding stress at germination and seedling stages [10, 11]. Therefore, comprehending the variation in flood tolerance among the genotypes and their underlying genetic architecture is important to develop an effective breeding strategy.

Over the past decades, extensive progress has been made in identifying essential parameters for assessing soybean flooding tolerance/susceptibility at germination or seedling stages [12–14]. Flooding is a complex quantitative trait influenced by multiple quantitative trait loci (QTLs)/genes with both major and minor effects and are largely influenced by the environment. Several QTLs for soybean flooding tolerance have been reported using bi-parental mapping populations [14–18]. So far, 27 QTLs have been detected and reported on SoyBase, which are scattered across the 20 chromosomes in the soybean genome, while several are recently detected [19, 20]. For instance, Dhungana et al. (2020) recently identified 20 QTLs with phenotypic variation explained (*R*^*2*^) and log of odd (LOD) in the range of 5.8–33.3% and 3.59–19.73%, respectively. Out of these, chromosomes 10, 12, and 13 harbored relatively more stable QTLs. However, most of the earlier reported QTLs were detected by linkage mapping strategy with several limitations, making them challenging to use for breeding program. Genome-wide association study (GWAS) provides more extensive ancestral recombination events due to high density of SNP markers. Therefore, to overcome the limitations associated with bi-parental mapping, GWAS has proven to be more effective and efficient in unraveling the genetic architecture of complex traits in soybean [21–25].

In soybean, there are only two independent studies on flooding tolerance with GWAS [26, 27]. In the study of Yu et al. (2019), 25 and 21 quantitative trait nucleotides (QTNs) were detected by the mixed linear model (MLM) and multi-locus random-SNP-effect mixed linear model (mrMLM) of GWAS, respectively. Out of these, Q*TN13* on chromosome Chr13 detected by the two distinct models for germination rate (GR), electrical conductivity (EC), and normal seedling rate (NSR). From Q*TN13,* one candidate gene, *Glyma.13 g248000* (*GmSFT*) was predicted, which was found to have a nonsynonymous mutation in the seed flooding tolerant genotypes, resulting in an amino acid alternation in the protein. Wu et al. (2020) examined flooding tolerance when more than 50% of plants were in the reproductive growth stage with a blooming flower. Fourteen SNPs distributed on 4 chromosomes viz., Chr03 (8), Chr04 (1), Chr07 (2), Chr13 (1) and Chr19 (2) were detected simultaneously by 4 single-locus models.

From the above, it is clear that less progress has been made in identifying genomic regions associated with soybean flooding tolerance, which is the first step toward identifying candidate genes. In order to speed up the genetic improvement of flooding tolerance in soybean, it is necessary to uncover the molecular mechanisms and develop gene-based functional markers suitable for marker-assisted selection (MAS). The present study used 243 diverse soybean plant introductions (PIs) obtained from the SoySNP50K BeadChip project available on SoyBase (http:www.soybase.org) [28] and utilized two distinct models of GWAS to identify SNPs linked to soybean seed flooding tolerance indices at the germination stage. In addition, putative candidate genes within the stable regions were predicted. The findings may be helpful for MAS to develop high-yielding and seed flood-tolerant soybean cultivars.

## Results

### Phenotypic variation and correlation among seed flooding tolerance parameters

To evaluate the phenotypic variation under seed flooding stress in the 243 soybean PIs accessions, the seeds were treated for 72 h under flooding stress and well-watered control. Four traits viz., electrical conductivity (EC), germination rate (GR), shoot length (ShL), and root length (RL) linked to seed flooding tolerance were measured at the germination stag. The descriptive statistics, *h*^2,^ and ANOVA of each trait for the 243 soybean accessions in 2018 and 2019 are presented in Table 1. The *h*^2^ for the four traits ranged from 0.78–0.99 (Table 1). EC was significantly affected by genotype (G), environment (E), and their interaction (GE) in the joint analysis. GR was influenced considerably by only G, whereas ShL and RL were affected by both G and GE (Table 1). There was a significant negative correlation coefficient (*r*^*2*^) with EC and the other three traits. On the other hand, significant positive *r*^*2*^ existed among the GR, ShL and RL (Table 1). The results revealed that these four traits are more under genetic control and can be used to screen and select for seed flooding tolerance.

**Table 1:** Statistics for seed flooding tolerance traits of 243 PI soybean accessions

Trait^a^	Env.^b^	Mean ± SD^c^	Min	Max	*h*^*2*d^	F values from ANOVA^e^			Pearson correlation		
						G	E	G × E	GR	ShL	RL
EC	2018	492.45 ± 294	120.00	1858.5	0.79	667.51***	21.67***	1.97***	- 0.40*	- 0.50*	- 0.50*
	2019	485.69 ± 295	106.05	1867.5							
GR	2018	0.76 ± 0.15	0.19	0.95	0.98	21.48***	0.24 ^ns^	3.24^ns^		0.36*	0.38*
	2019	0.76 ± 0.14	0.23	0.97							
ShL	2018	0.78 ± 0.15	0.27	0.99	0.98	30.94***	0.88^ns^	2.17***			0.73*
	2019	0.79 ± 0.14	0.26	0.99							
RL	2018	0.72 ± 0.20	0.2	0.98	0.99	35.26***	0.78^ns^	5.05***			
	2019	0.72 ± 0.19	0.24	0.98							

^a^*EC* Electrical conductivity (μS·cm^−1^/g^−1^), *GR* Germination rate, *ShL* Shoot length (cm), *RL* Root length (cm)

^b^Environment

^c^Average of 2 biological repeats ± standard deviation

^d^Broad-sense heritability

^e^G-Genotype; E-Environment; GE-Genotype by Environment interaction

^***^indicates significance level at *p* < 0.001; ns indicates non-significant

^*^correlation is significant at the 0.05 level (2-tailed)

### Distribution of SNPs, population structure and linkage disequilibrium decay

**Fig. 1:**
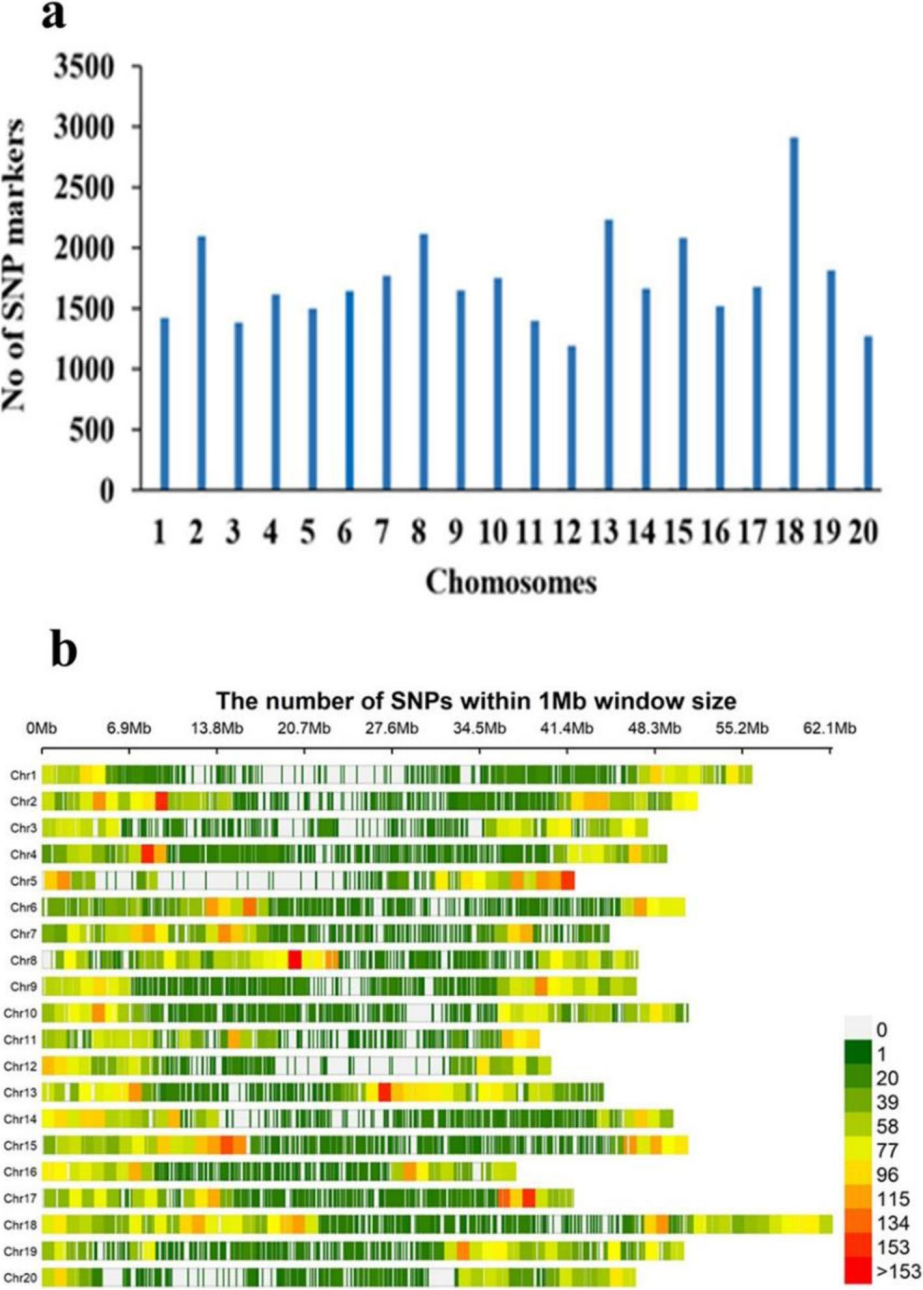
Distribution analysis of 34,718 SNPs across 243 soybean PIs. **a** Distribution of SNPs on 20 chromosomes. **b** The density of SNPs on each chromosome. The horizontal axis shows chromosome length (Mb); the vertical axis gives the chromosome number and the different colors depict SNP density (the number of SNPs per window)

**Fig. 2:**
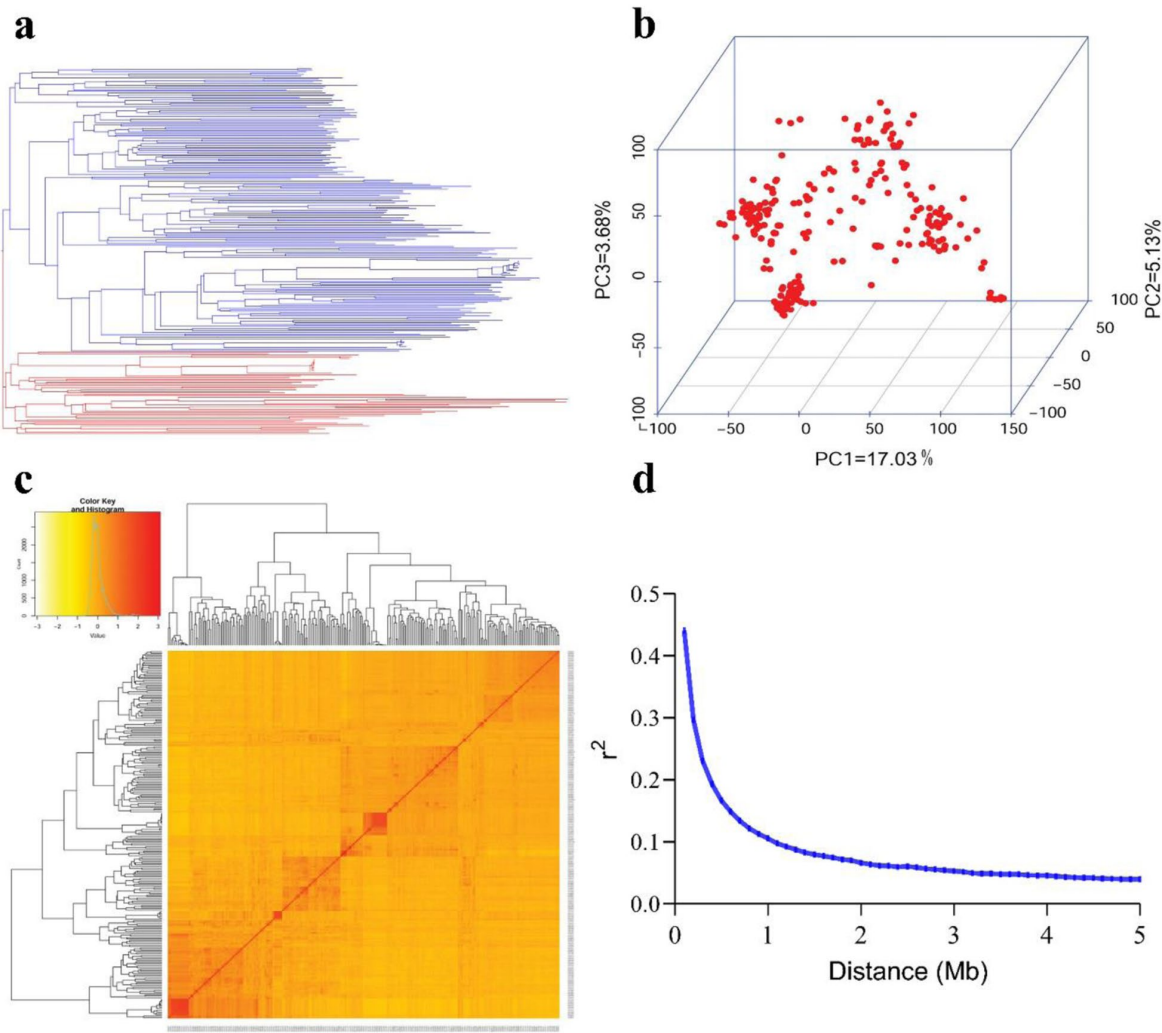
Genetic features of the mapping population. **a** A maximum likelihood neighbor-joining tree of the tested 243 lines. **b** Population structure of soybean germplasm. **c** A heatmap of the kinship matrix of the 243 soybean PIs accessions. **d** LD decay of the genome-wide association study (GWAS) population

A total of 243 soybean PIs accessions were genotyped with 42,449 SNP markers; however, 34,718 SNPs with MAF ≥ 0.05 were used for further analyses. These SNPs were found on 20 chromosomes with an average of 1736 SNPs on each chromosome (Chr) with a maximum (2912) on the Chr18 and a minimum (1192) on Chr12 (Fig. 1a). Chr13 had the highest SNP marker density (50.49 SNPs/Mb), and Chr01 had the least marker density (11.84 SNPs/Mb) (Fig. 1b). In the present study, the population structure obtained by phylogenetic analysis of 34,718 SNPs divided 243 accessions into 2 clusters (Fig. 2a). The first three principal components (PCs) accounted for 25.84% of the total genetic variation (Fig. 2b). Based on the distribution of the pairwise relative kinship coefficients, the 243 tested PI accessions had a lower level of genetic relatedness, which aligned with the phylogenetic tree (Fig. 2c). Furthermore, *r*^*2*^ declined with increasing distance, and the average LD decay distance by 34,718 SNPs for LD analysis was about 400 kb, with *r*^*2*^ dropping to half of its highest value (Fig. 2d).

### GWAS analysis with two distinct models

**Fig. 3:**
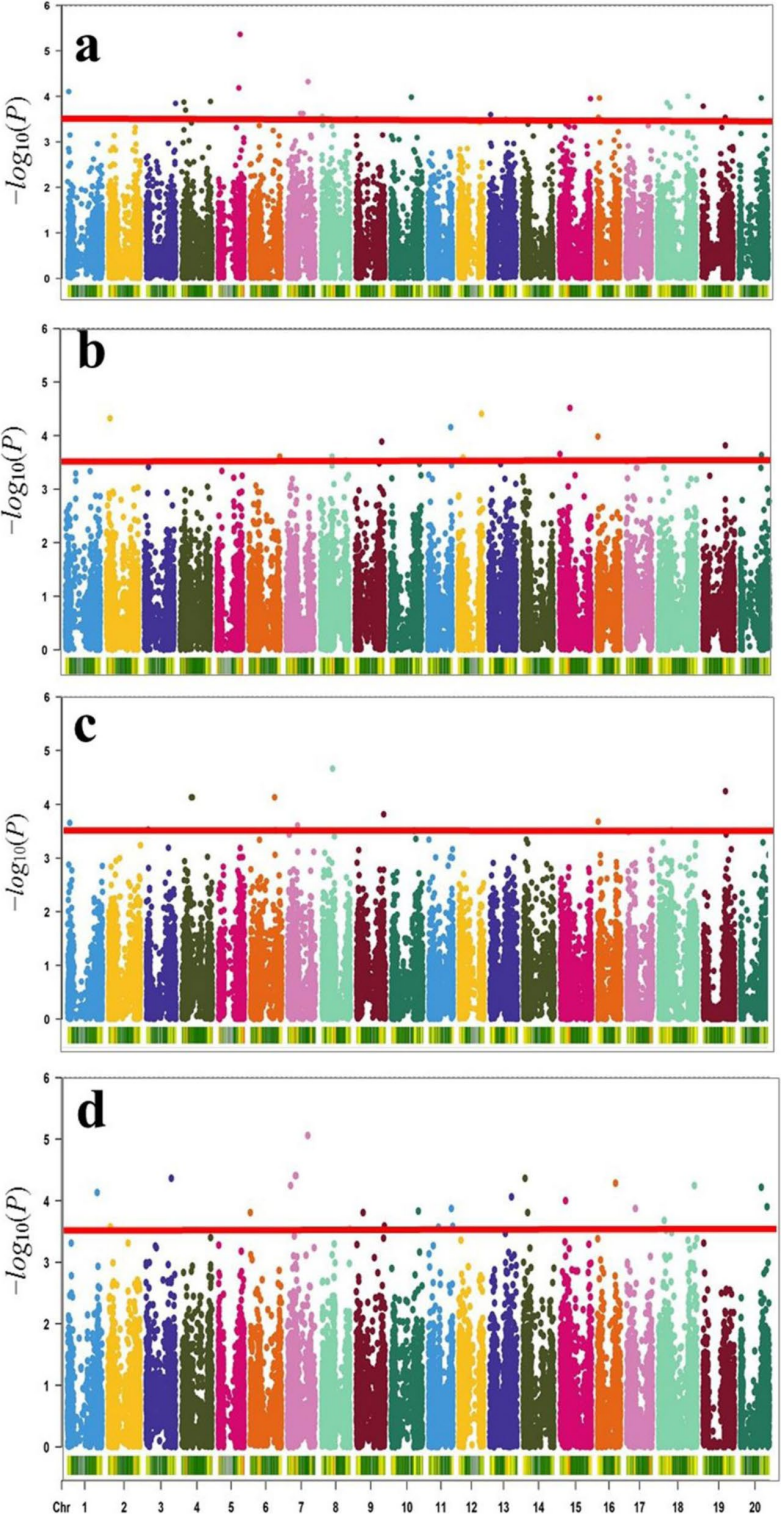
Manhattan plots for GWAS of the 243 soybean accessions for Electrical conductivity (EC) (**a)**; Germination rate (GR) **(b)**; Shoot length (ShL) **(c)**; Root length (RL) **(d)** using MLM (PCA + K). The threshold of 3.5 was adopted with a red line in the Manhattan plots. The X-axis represents chromosome number and Y-axis represents −*log*_10_(*P*)

Two models comprising one single-locus model (MLM) and one multi-locus model (mrMLM) were used to perform SNP-trait association to identify significant SNP associated with EC, GR, ShL and RL. In MLM with a threshold of -*log*_*10*_*(p)* ≥ 3.5, a total of 20 SNPs were significantly associated with EC in 2018, 2019, plus the average across two environments on 4 chromosomes viz. Chr04, Chr08, Chr14, and Chr17 (Additional File 1: Table S1; Fig. 3a-d). We also identified 40, 4, and 9 SNPs associated with GR, ShL, and RL, respectively, via MLM in all environments (2018, 2019 and with the average of the 2 years) (Additional File 1: Table S1; Fig. 3a-d). Out of these, Gm_08_11971416 which associated with EC (*log*_*10*_*(P)* = 5.16) and GR (*log*_*10*_*(P)* = 5.06) met the Bonferroni correction criteria (≈ 4.54). SNPs related with GR Gm_08_46236506, Gm_08_46239716, and Gm_08_46242569 met the Bonferroni correction criteria as well (Additional File 1: Table S1). A total of 27, 17, 13, and 18 SNPs were detected by mrMLM for EC, GR, ShL, and RL traits, respectively, in the individual and joint environments (Additional File 2: Table S2). These SNPs were detected on all chromosomes with the exception of Chr06 and Chr17 with *R*^*2*^ (2.27–12.13%) and LOD (3.01–9.24, thus, −*log10(P)* = 3.71–10.17) (Additional File 2: Table S2).

**Fig. 4:**
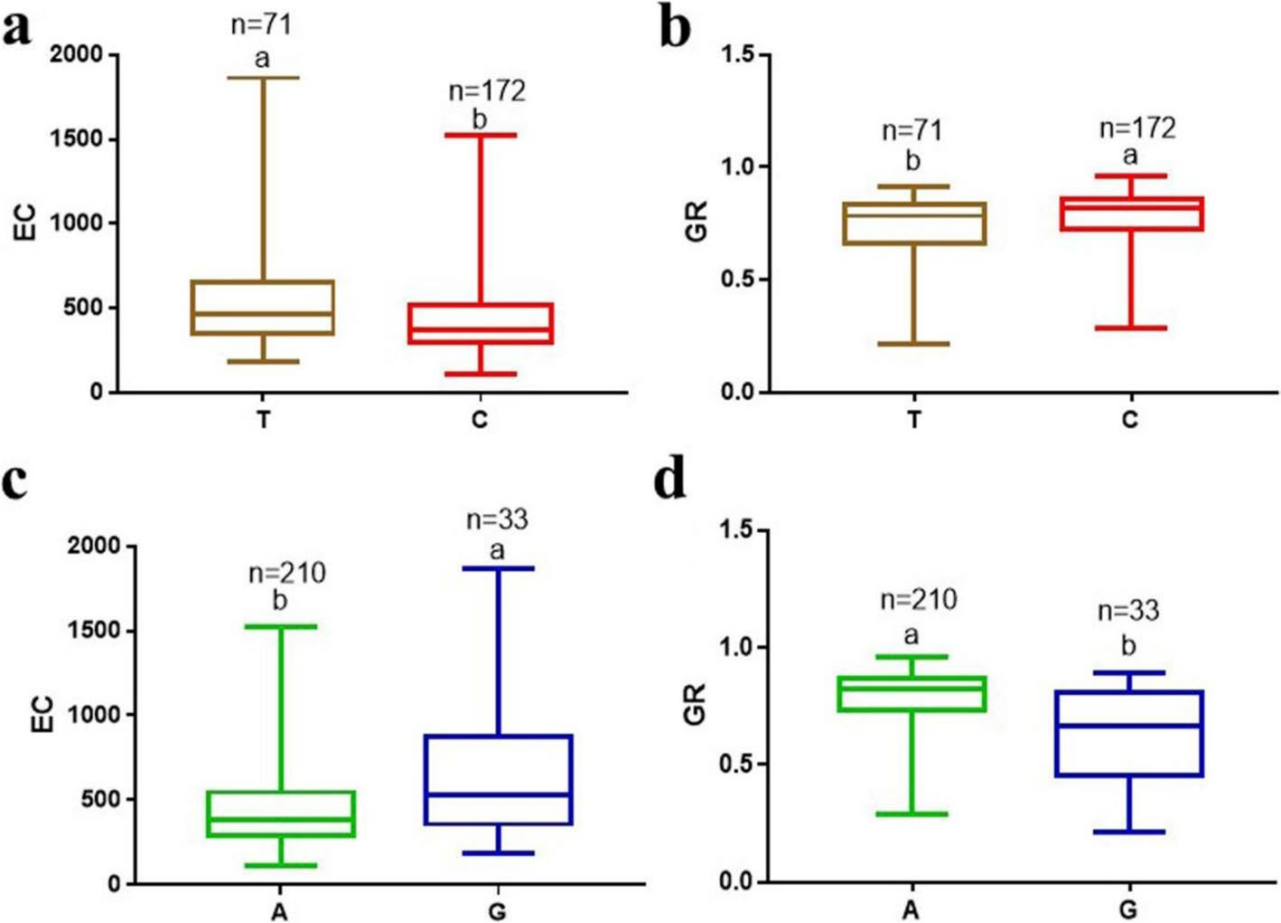
Effect of alleles of Gm_08_11971416_C/T) and Gm_08_46239716_ A/G) on electrical conductivity (EC) expressed as μS·cm^− 1^ g^− 1^ and Germination rate (GR). **a** C/T on EC. **b** C/T on GR. **c** A/G on EC. **d** A/G on GR. The line in the middle of each box represents mean. The boxes in each plot with different alphabets on top indicate significant difference with Post-hoc means separation by Duncan Multiple Range Test at 5% probability. The *n* represents the number of accessions with the allele in focus

In the comparison of results from MLM and mrMLM, two major SNPs (Gm_08_11971416 and Gm_08_46239716) on Chr08 were detected concurrently for EC and GR (Table 2). In the same direction, Gm_08_11971416 was found to be associated with ShL and RL. The alleles at Gm_08_11971416 (C/T) and Gm_08_46239716 (A/G) caused significant variation in EC and GR (Fig. 4a-d). Moreover, Gm_05_1000479 and Gm_01_53535790 SNPs were identified for ShL and RL, respectively, in both models (Table 2). These four major SNPs on Chr01, Chr05, and Chr08 were considered as major and stable loci and used for mining potential candidate genes underlying seed-flooding tolerance related traits in this study.

**Table 2:** Four SNPs associated with electrical conductivity (EC), germination rate (GR), shoot length (ShL), and root length (RL) on MLM and mrMLM models

Traits ^a^	SNP ID^b^	Chr^c^	Position (bp)	Allele	MLM^d^		mrMLM^f^	
					*−log*_*10*_ *(p)*	*R*^2^ (%)^e^	LOD^g^	*R*^2^ (%)^e^
EC	Gm_08_11971416	8	11,971,416	C/T	5.16–5.25	8.80–8.99	7.99–9.25	7.78–9.20
	Gm_08_46239716	8	46,239,716	A/G	3.98–4.08	6.48–6.66	3.78–5.53	4.88–7.26
GR	Gm_08_11971416	8	11,971,416	C/T	4.82–5.08	8.29–8.84	4.93–5.84	5.19–6.89
	Gm_08_46239716	8	46,239,716	A/G	4.88–5.79	8.26–10.12	7.14–7.24	9.54–12.13
ShL	Gm_05_1000479	5	1,000,479	A/C	3.73–4.31	6.08–6.87	7.38	9.92
RL	Gm_01_53535790	1	53,535,790	A/G	3.78–3.88	6.09–6.28	3.69	6.07

^a^*EC* Electrical conductivity (μS·cm^−1^/g^−1^), *GR* Germination rate, *ShL* Shoot length (cm), *RL* Root length (cm)

^b^*Single* nucleotide polymorphism

^c^Chromosome

^d^Mixed Linear Model

^e^Phenotypic variation explained (%)

^f^*multi-locus random-SNP-effect mixed linear model (mrMLM*)

^g^logarithm of odds

### Allele effect of SNPs associated with seed flooding tolerance

**Fig. 5:**
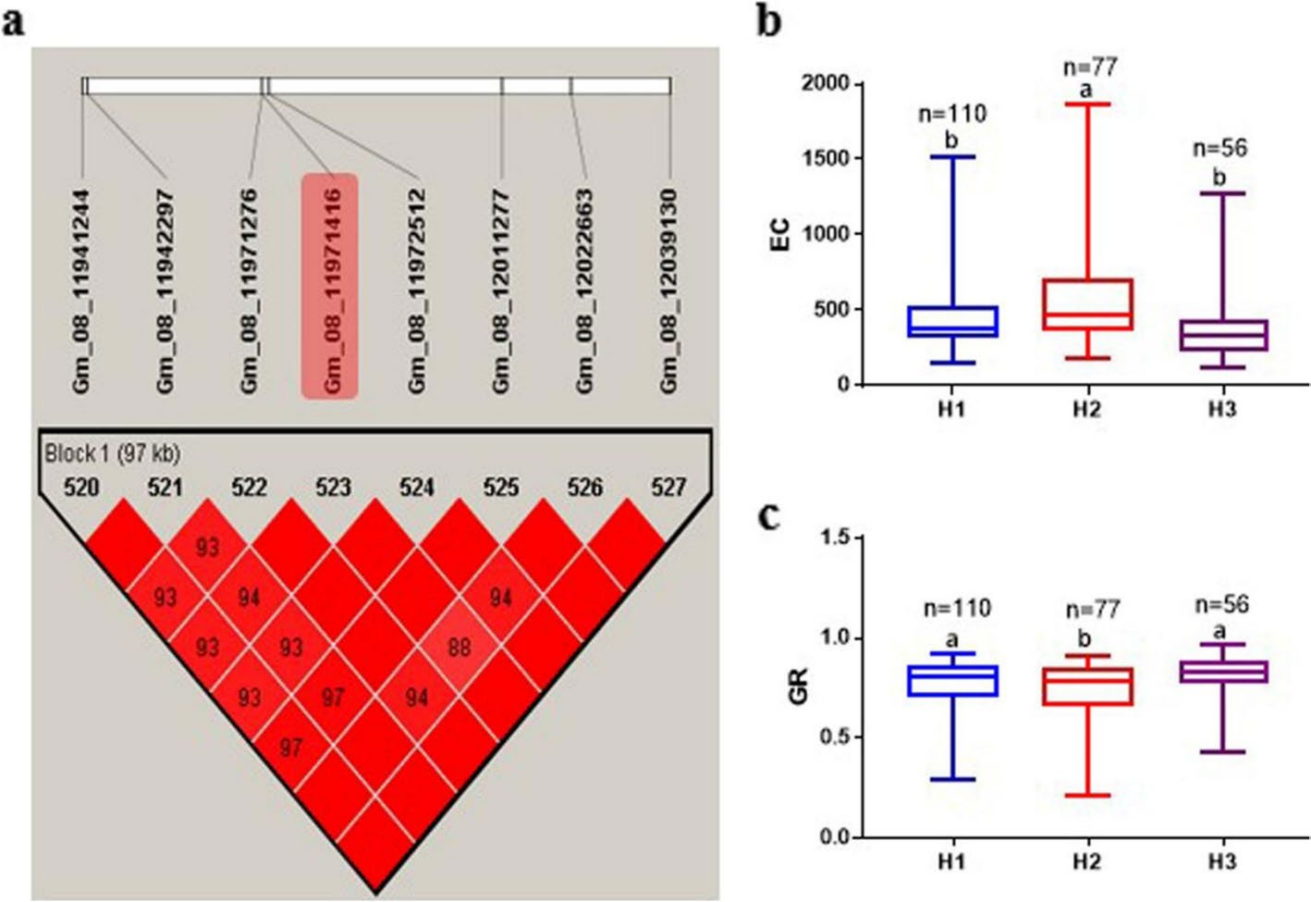
Haplotype block around Gm_08_11971416 and phenotypic variation analysis. **a** Haplotype block. **b** Haplotype groupings and their variation in electrical conductivity (EC, expressed as μS·cm^− 1^ g^− 1^). **c** Haplotype groupings and their variation in germination rate (GR). Groupings H1, H2 and H3 have TCTCATCC, TCCTGTCC and CTTCACTA, respectively. The line in the middle of each box represents mean. The boxes in each plot with different alphabets on top indicate significant difference with Post-hoc means separation by Duncan Multiple Range Test at 5% probability. The *n* represents the number of accessions with the alleles combined

**Fig. 6:**
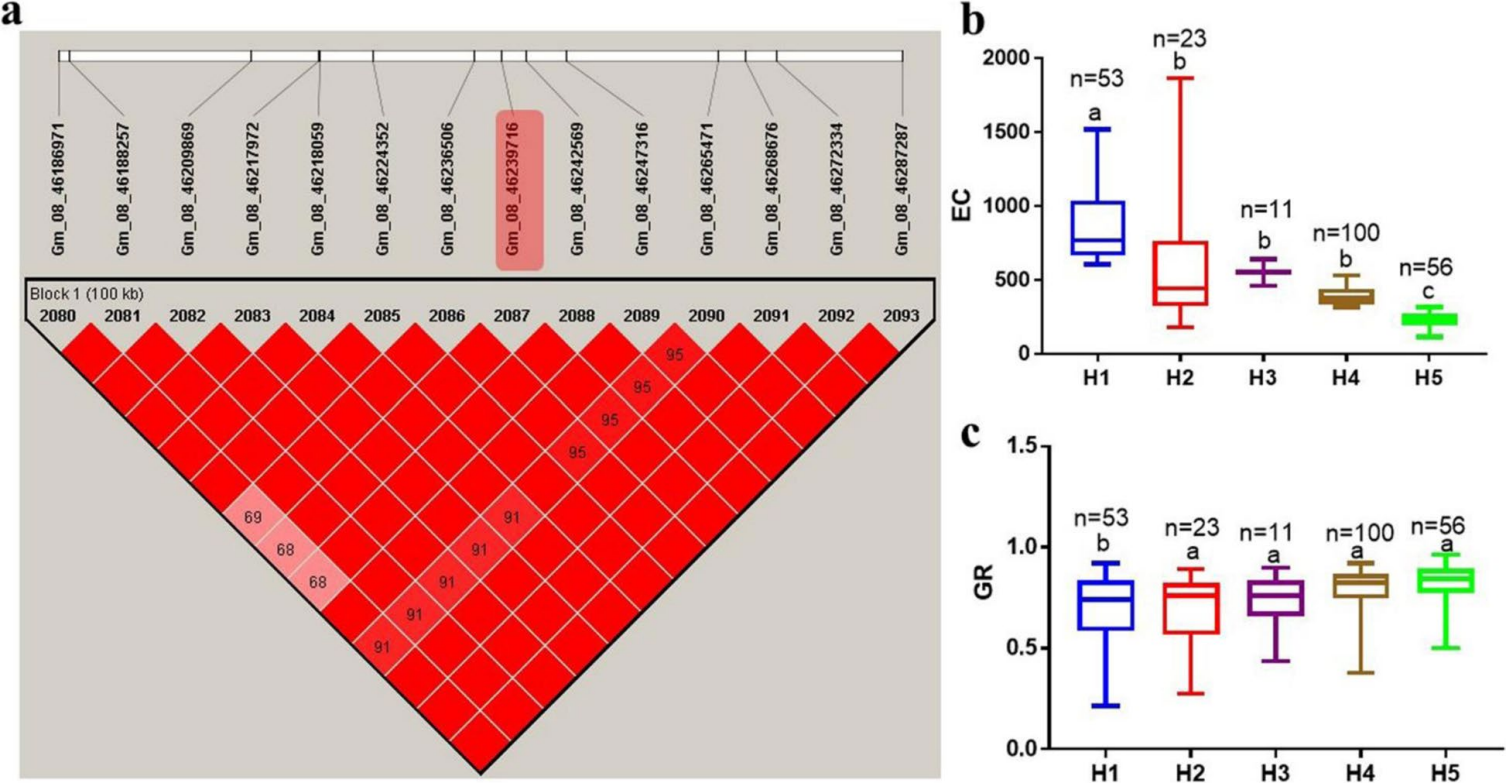
Haplotype block around Gm_08_46239716 and phenotypic variation analysis. **a** Haplotype block. **b** Haplotype groupings and their variation in electrical conductivity (EC, expressed as μS·cm^− 1^ g^− 1^). **c** Haplotype groupings and their variation in germination rate (GR). Groupings H1, H2, H3, H4 and H5 have AGGTAAATCTATT, CAAGCCGCTCCCC, CAAGCAATCTATT, CAAGCCGCCTATT, and AGGTCAATCTATT, respectively. The line in the middle of each box represents mean. The boxes in each plot with different alphabets on top indicate significant difference with Post-hoc means separation by Duncan Multiple Range Test at 5% probability. The *n* represents the number of accessions with the alleles combined

Haplotype block analyses were performed for Gm_08_11971416 and Gm_08_46239716 SNPs associated with EC and GR using Haploview 4.2 software. The distance within each block ranged 97–100 kb with range of 8–14 SNPs. The 243 soybean PIs accessions were grouped into three haplotypes (H1-H3) around Gm_08_11971416 (Fig. 5a). The mean ± standard error values of EC for H1, H2 and H3 varied significantly (*P < 0.05*), thus, 476.60 ± 25.46, 594.1 ± 39.25, and 365.4 ± 28.26 μS·cm^− 1^ g^− 1^, respectively (Fig. 5b). Similarly, groupings of the accessions on the basis of GR differed significantly (0.77 ± 0.01, 0.72 ± 0.02 and 0.81 ± 0.01 for H1, H2 and H3, respectively) (Fig. 5c). On the other hand, haplotype block around Gm_08_46239716 divided the 243 accessions into five groups (H1-H5) (Fig. 6a-b). The EC and GR of these groups differed significantly (*P < 0.05*). The highest EC was observed in H1 (873.7 ± 35.11 μS·cm^− 1^ g^− 1^), while H3 had the least EC (226.3 ± 7.25 μS·cm^− 1^ g^− 1^) (Fig. 6b). In terms of GR, the highest and least were observed in H5 (0.83 ± 0.01) and H2 (0.68 ± 0.04), respectively (Fig. 6c).

### Candidate and hub genes prediction underlying seed flooding tolerance

**Fig. 7:**
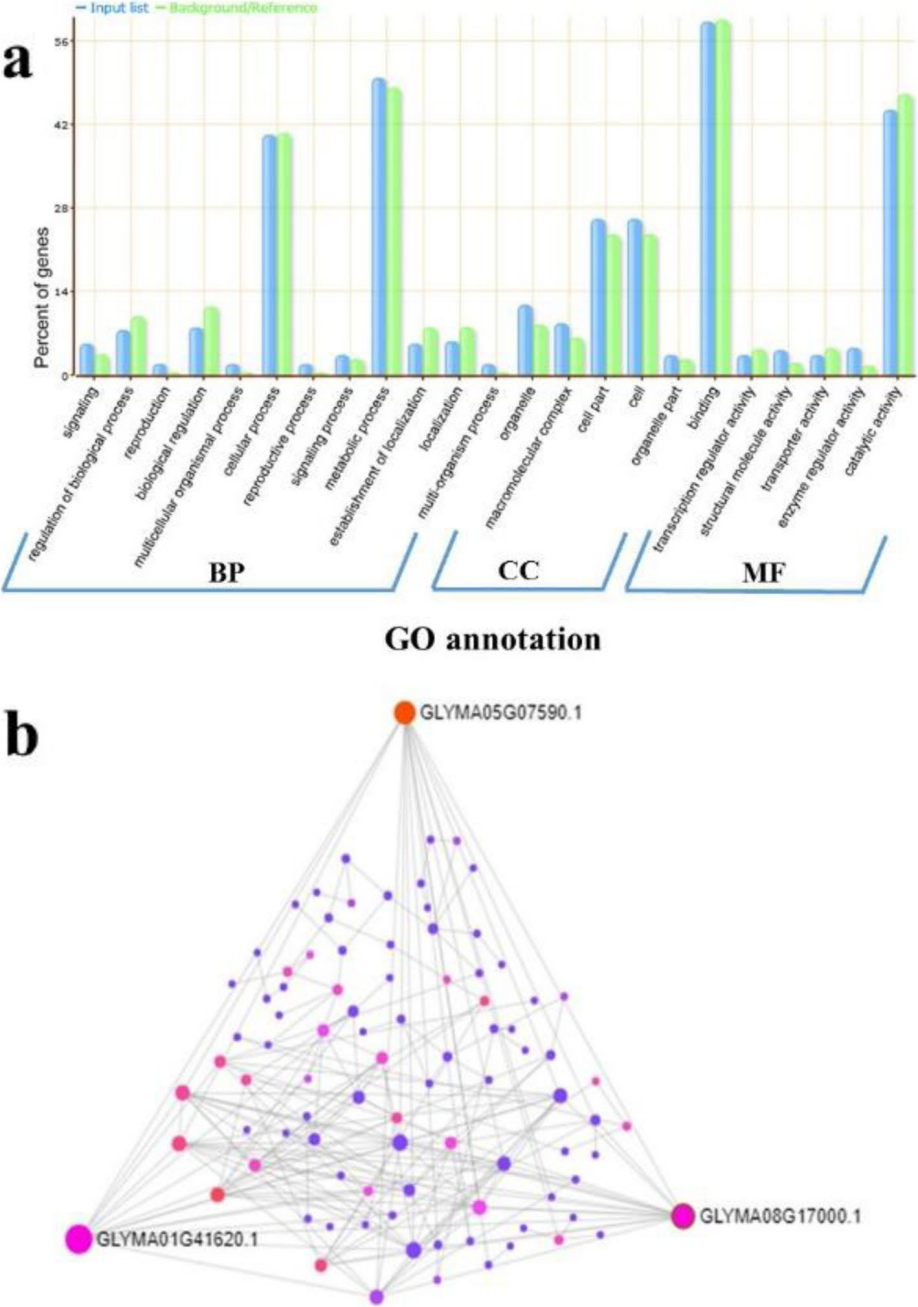
Candidate gene analysis. **a** Diagram showing Gene Ontology (GO) enrichment analysis for Biological processes (BP), Cellular components (CC), and Molecular function (MF) using agriGO toolkit **(b)** Graphical depiction of the selective hub module of sub-network-1. Hub genes are highlighted in red and pink. Edges of the hub are shown in gray

Based on the physical position of four SNPs viz., Gm_08_11971416, Gm_08_46239716, Gm_05_1000479, and Gm_01_53535790 linked with seed flooding tolerance, we performed the candidate gene prediction analysis with the SNP position ±500 kb. A total of 483 model genes were found within the regions of the four SNPs using Glyma2.0 models in SoyBase (Additional File 3: Table S3-S6). To further clarify the potential functions of these genes, various functional groups were categorized based on GO enrichment analysis (http://bioinfo.cau.edu.cn/agriGO). Of these genes, 61 had no functional annotations (representing NA) (Additional File 3: Table S3-S6). A total of 422 genes were assigned to one of the three GO categories: biological processes (BP), molecular function (MF) and cellular components (CC). The highest percentage of genes were connected with the GO terms cellular process (GO:0009987), metabolic process (GO:0008152), cell part (GO:0044464), cell (GO:0005623), binding (GO:0005488) and catalytic activity (GO:0003824) (Fig. 7a). Hence, these GO terms may play roles in regulating mechanisms in soybean seed-flooding stress.

The annotated 422 genes were related to plant growth regulation, flavonoids biosynthesis, phytohormone signaling pathways, stress signal perception and transduction, seed germination, flowering and senescence, embryo development, secondary wall synthesis, and plant hormonal responses, such as gibberellin responses, and ethylene biosynthesis (Additional File 3: Table S3-S6). By considering the results of GO enrichment analysis as well as gene functional annotations from the SoyBase, and Phytozome databases, we predicted eight stress responsive genes, *Glyma.01G198000* (Transcription factor bHLH18), *Glyma.01G206300* (G-Type Lectin S-Receptor-Like Serine/Threonine-Protein Kinase), *Glyma.05G006700* (Protein kinase superfamily protein) *Glyma.05G008000* (CCCH-type zinc finger family protein), *Glyma.08G152800* (Leucine-rich repeat family protein), *Glyma.08G152900* (Tetratricopeptide repeat like superfamily protein), *Glyma.08G344500* (GATA Transcription factor 26), and *Glyma.08G348500* (UDP-glycosyltransferase) from the 422 genes (Table 3). The results suggest that flood*-*response mechanisms in soybean are complex and may be governed by several signal pathways.

**Table 3:** A subset of candidate and hub genes adjacent four SNPs associated with soybean seed flooding tolerance at the germination stage

Candidate Gene ID ^**a**^	Position (bp)^**b**^	Homolog in Arabidopsis	Functional annotation^**c**^
*Glyma.01G198000*	53,200,980–53,202,684	*AT4G37850*	Transcription factor bHLH18
*Glyma.01G206300*	53,862,540–53,862,540	*AT5G60900*	G-Type Lectin S-Receptor-Like Serine/Threonine-Protein Kinase
*Glyma.05G006700*	608,574–621,690	*AT5G14720*	Protein kinase superfamily protein
*Glyma.05G008000*	749,837–753,271	*AT2G02160*	CCCH-type zinc finger family protein
*Glyma.08G152800*	11,760,404–11,762,319	*AT3G12610*	Leucine-rich repeat (LRR) family protein
*Glyma.08G152900*	11,780,677–11,784,610	*AT5G40410*	Tetratricopeptide repeat (TPR)-like superfamily protein
*Glyma.08G344500*	45,957,501–45,962,417	*AT4G17570*	GATA Transcription factor 26
*Glyma.08G348500*	46,292,645–46,301,440	*AT2G15490*	UDP-glycosyltransferase
**Hub genes**			
*Glyma.01G207700*	53,959,688–53,961,931	*AT4G16720*	Ribosomal protein L23/L15e family protein
*Glyma.05G016800*	1,491,261–1,493,536	*AT5G08180*	Ribosomal protein L23/L15e family protein
*Glyma.08G159800*	12,386,775–12,390,398	*AT3G04770*	40s ribosomal protein SA B

^a^Genes position in Glyma2.0 version obtained from SoyBase while those with bolded font are hub genes (highly connected genes in the network)

^b^Genes start and end positions in Glyma2.0 version

^c^Possible functional annotation obtained from SoyBase (https://soybase.org/) and Phytozome (https://phytozome.jgi.doe.gov/pz/portal.html)

To construct possible protein-protein interaction (PPI) networks associated with seed flooding tolerance, we used STRING online software. The possible network obtained from STRING was subsequently visualized in the Network Analyst platform. From PPI network analysis, we identified three hub genes viz., *Glyma.01G207700* (Ribosomal protein L23/L15e family protein), *Glyma.05G016800* (Ribosomal protein L23/L15e family protein) and *Glyma.08G159800* (40s ribosomal protein SA B) that may be associated with soybean seed-flooding tolerance (Fig. 7b).

We retrieved RNA-Seq data of these candidate genes from SoyBase (www.soybase.org). Based on RNA-seq analysis, all the predicted candidate genes showed significantly higher gene expression in the root tissues, root nodule, young leaf, flower, and pod shell as well as seed developmental stages except *Glyma.08G344500* (Additional File 4: Figure S1). In addition, the predicted candidate and hub genes were found to possess phytohormone *Cis-*elements such as auxin-responsive element (TGA-element and AuxRR-core), jasmonic acid (CGTCA-motif), abscisic acid (ABRE), salicylic acid (TCA-element) and others (Additional File 5: Table S7). Hence, these candidate and hub genes might be associated with soybean seed-flooding tolerance. However, they need further functional validation to check their actual roles in seed flooding tolerance.

## Discussion

### Genetic variation and correlation of traits evaluated for seed flooding tolerance in soybean

Seed flooding stress is a severe abiotic stress that reduces soybean seed yields by affecting seed germination, seedling growth, and development [27, 29]. Since seed-flooding tolerance is a complex quantitative trait, it is imperative to understand the genetic basis and genes involved in seed-flooding tolerance and this has been a major focus in soybean for a breeding program targeted at developing flood-tolerant cultivars. To date, the genetic mechanisms controlling seed flooding tolerance in soybean are not well understood at the germination stage. Seed germination is governed by different phytohormones and environmental conditions [30]. The strong correlation between seed-flooding tolerance and its related traits, viz., EC, GR, NSR, ShL, and RL in soybean has been previously reported [12–14, 26, 31]. Therefore, we examined EC, GR, ShL, and RL as the most important determinants to better estimate soybean seed-flooding tolerance. These traits are primarily regulated by genetic factors, as shown in ANOVA (Table 1) which are in consonance with earlier reports [27, 31]. According to a previous study, seed flooding stress caused seed material leakage and subsequent seed damage due to rapid water imbibition, which was confirmed by EC measurements [14]. As a result, we examined the EC of 243 soybean PIs accessions to better assess soybean seed-flooding tolerance. Furthermore, significant positive correlations among GR, ShL, and RL were observed in the soybean PIs accessions, and EC had a negative correlation with GR, ShL, and RL (Table 1). Overall, the comprehensive analysis of EC, GR, ShL, and RL is an important consideration in determining seed-flooding tolerance during germination in soybean.

### Population structure and linkage disequilibrium in the panel

As evident from the phylogenetic tree and kinship plot, the population structure divided the 243 lines into 2 sub-groups (Fig. 2a, c). The sub-group 1 (blue) comprised mainly accessions from United States, Brazil, and Zimbabwe etc. whereas sub-group 2 (red) consisted accessions from mainly from Asia (China, Nepal, Bhutan, India, and Vietnam etc.). This is not surprising as soybean is noted to have originated and domesticated in East Asia [32]; thus, possibly the accessions used may have their lineage from Asia, specifically China. These findings pinpoint the grouping of accessions using molecular markers that showed a sub-structure based on the geographical origin of accessions. The extent of LD is a crucial determinant of association analysis efficiency [33]. In this study, the LD decay of the soybean genome was estimated to be about 400 kb which was considerably higher than that of other plants like rice (75 kb in *indica* rice) [34] and *Arabidopsis thaliana* (10 kb) [35]. This is related to the cleistogamous features of soybeans, which might have a significant impact on genomic homogeneity and lower genomic variation, and this character may become more sensitive to domestication practices due to low genetic diversity and high LD [36, 37].

According to previous studies, there are 27 QTLs associated with flooding tolerance in the soybean genome, distributed across 15 chromosomes, suggesting that flooding tolerance is a complex quantitative trait regulated by several genes with both substantial and modest impacts in soybean.

### Significant SNPs and previously reported regions

Limited QTL mapping studies have been undertaken to detect the genomic regions linked with flooding tolerance using different bi-parental mapping populations. According to previous studies, there are 27 QTLs associated with flooding tolerance in the soybean genome, distributed across 15 chromosomes, suggesting that flooding tolerance is a complex quantitative trait and regulated by multiple genes with both major and minor effects in soybean [14–18]. However, most of these QTL mapping studies were conducted using low-density genetic maps based on linkage mapping. GWAS leverages on LD in the natural population and helps to overcome the limitations in linkage mapping. Hence, the present study used diverse soybean germplasm from 22 different countries to perform two distinct models of GWAS. There are numerous single-locus models, however MLM is more popular possibly because of its accounts of population structure and familial relatedness among the study population to avoid possible spurious marker-trait associations [38, 39]. However, the single-locus models are limited in detecting marginal effects QTNs influenced by the polygenic background and stringent Bonferroni correction [40]. For example, out of the numerous SNPs studied, only Gm_08_11,971,416 connected to EC and GR, Gm_08_46236506, Gm_08_46239716, and Gm_08_46242569 linked to GR met the Bonferroni correction criteria (≈ 4.54). Therefore, we adjusted the threshold in MLM to > 3.5 [41, 42]. Briefly, it has been established that a Bonferroni threshold (example *P =* 2.88E-05 or -log_10_(1/34,718) = 4.54, in the current study) is overly strict when the linkage disequilibrium among genetic markers is large, which is generally the case with soybean [43, 44]. Therefore, we adjusted to *P* ≤ 0.0003 or -log_10_(*P*) > 3.5 in the current study was used, which is less stringent than the Bonferroni-corrected threshold, but more stringent than the threshold used in the study by Wu et al. [27] (−log_10_(*P*) > 2.5) and Song et al. [45] (−log_10_(*P*) > 3.0) with accessions and SNPs from SoySNP50K BeadChip. The adopted threshold in study was similar to Kaler et al. (2017) who utilized 373 accessions and 31,260 SNPs and Chamarthi et al. (2021) with 200 accessions and 34,680 SNPs from SoySNP50K BeadChip to map genomic regions associated with soybean canopy wilting.

To overcome the limitation in MLM, we implemented mrMLM (Multi-locus model) with the LOD = 3 which has been recommended in multi-locus GWAS to balance high power and low false positive rate [46]. Multi-locus GWAS has been developed as a multidimensional genome scan method in which the effects of all markers are estimated at the same time [47]. mrMLM is emerged as a powerful tool for genetic dissection of complex traits [40].

Previous studies reported that GWAS is an effective and efficient strategy for detecting the genetic loci and candidate genes associated with complex quantitative traits [23, 48, 49]. MLM effectively controls genomic inflation and is widely used in genome-wide association analysis to identify QTL associated with soybean traits [50–52]. In the present study, a total of 73 and 75 significantly-associated SNPs of EC, GR, ShL, and RL traits were detected by MLM (Additional File 1: Table S1) and mrMLM, respectively (Additional File 2: Table S2). Among these SNPs, two major and stable SNPs, Gm_08_11971416 and Gm_08_46239716, were detected across the environments for EC and GR traits at the significance threshold *-log*_*10*_ *(P)* ≥ 3.5 (Table 2). In addition, Gm_05_1000479 and Gm_01_53535790 SNPs were identified for ShL and RL, respectively, in both models.

The stability of QTL is a requisite for their use in a practical breeding program such as MAS. Therefore, the major and stable SNPs (Gm_01_53,535,790, Gm_05_1,000,479, Gm_08_11,971,416, and Gm_08_46,239,716) (Table 2) could further be validated and exploited further for their use in the breeding program. Also, some associated SNPs in this study overlapped with a number of earlier reported regions; Gm_01_3260769 linked to ShL detected by mrMLM co-located with the physical region of *Flood tolerance 6–1* (1031087–7,729,201 bp) [53]. Similarly, SNP at 10789902 bp overlapped with *Flood tolerance 7–2* [14] on Chr08. Also, two SNPs; Gm_13_20995641 and Gm_14_4550573 co-located within the genomic regions of *Flood tolerance 6–3* [53] and *Flood tolerance 4–5* [16]. All the SNPs detected on Chr17 except for Gm_17_8735403 co-located within a recent QTL (*qSFT_17–52*) reported by Dhungana et al. [19]. Last but not the least, *Flood tolerance 6–2* [53] harbored 2 SNPs on Chr19 in this study (Gm_19_40771881 & Gm_19_40783256). These results give credence to the findings of this study; however, the new SNPs/loci detected need further verification for their use in the breeding program. Also, the haplotype alleles detected on the major loci on Chr08 (Figs. 5 and 6) could be exploited for haplotype-based breeding [54, 55].

### Candidate genes analysis

It is of great interest to identify the candidate genes underlying the genomic region for practical plant breeding. To date, the SoyBase has reported on 27 QTLs related to soybean flooding tolerance. However, none was used to mine putative candidate genes with the exception of recent studies [26, 27]. For example, *GmSFT* (*Glyma.13 g248000*) showed significantly higher expression levels in tolerant genotypes than sensitive genotype under seed flooding stress [27]. In this study, candidate genes were predicted in the genomic region surrounding four major and stable SNPs, viz., Gm_08_11971416, Gm_08_46239716, Gm_05_1000479, and Gm_01_53535790 (Table 2). Most of the genes are related with the terms cellular process, metabolic process, cell part, cell, binding, and catalytic activity as per GO enrichment analysis, and these GO terms are reported to be essential in seed flooding tolerance mechanisms [56].

Based on the GO enrichment analysis, and gene functional annotations we predicted eight genes as the possible candidate genes (Table 3). The gene *(Glyma.01G198000)*, located on Chr01, encodes a basic helix-loop-helix (bHLH) transcription factor that is involved in plant adaptive responses to various abiotic stresses, including drought, salinity, heavy metal stress, oxidative stress, iron deficiency, low temperature stresses, and osmotic stress [57–59]. On Chr01, there is also a gene *(Glyma.01G206300)* that codes for G-Type Lectin S-Receptor-Like Serine/Threonine-Protein Kinase and plays a crucial role in plant response to salt stress [60]. Gene *(Glyma.05G006700)* encodes a protein kinase that is involved in plant responses to drought, salt, and cold stress [61]. Gene *(Glyma.05G008000)* located on Chr05, encodes a CCCH-type zinc finger protein. *CCCH* genes contribute to seed germination by regulating abscisic acid (ABA), light and gibberellic acid in Arabidopsis [62]. The previous study in rice reported that *OsCCCH-Zn-1* is induced under hypoxia, submergence, and drought stresses [63]. On Chr08, *Glyma.08G152800* encodes a leucine-rich repeat protein. Overexpression of the leucine-rich repeat receptor-like kinase gene (*LRK2*) increased drought tolerance in rice [64]. Gene *(Glyma.08G152900)* is also located on Chr08 which encodes for a tetratricopeptide repeat like protein (TPR) involved in the plant hormonal regulation, such as ethylene biosynthesis, gibberellic acid and cytokinin responses [65, 66]. TPR proteins have also been found to be involved in the regulation of ABA signaling and abiotic stress responses [67]. The recent study demonstrated that *AtTPR10* functions as a molecular chaperone to protect plants from diverse abiotic stresses, such as low temperature, drought, and salinity [68].

Gene (*Glyma.08G344500*) encodes a GATA Transcription factor 26 that is involved in the regulation of growth processes and various environmental stresses, including salinity and drought [69]. A recent study by Zhao et al. (2021) revealed that overexpression of *SlGATA17* (*Solyc05g056120.2.1*) enhances drought tolerance with increased activation of the phenylpropanoid biosynthetic pathway in transgenic tomato compared to the wild type [70]. In addition, *Glyma.08G348500* encodes a UDP-glycosyltransferase that is involved in the regulation of grain size and multiple abiotic stress tolerance (salinity, drought and heat stress) in rice [71]. Furthermore, our study identified three hub genes (*Glyma.01G207700*, *Glyma.05G016800*, and *Glyma.08G159800*) as ribosomal protein genes in the functional co-expression network generated by network analyst that might regulate abiotic stress tolerance (Table 3). Previous studies reported that ribosomal protein genes enhance tolerance to drought, and heavy metal stress [72–74]. From the available RNA-seq data, the predicted candidate and hub genes expressed significantly higher gene expression in the various tissues and developmental stages, giving indication of their possibility of regulating soybean response to seed-flooding tolernace [75]. Hub genes (highly connected genes) are reported to modulate expression of large number of genes in a functional network of genes [23, 76]. In addition, with exception of *Glyma.08 g152900*, all the predicted candidate and hub genes contain *Cis-*elements which are essential in regulating plant response under flooding/waterlogging conditions (Additional File 5: Table S7). For example*, Glyma.01 g198000*, *Glyma.01 g206300* and *Glyma.05 g006700* contain auxin responsive element. It has been demonstrated that auxin accumulation in the stem triggers additional ethylene synthesis which stimulates a flux of auxin towards flooded parts of the plants [77, 78]. In addition, auxin accumulation in the base of the plant induces growth of pre-formed root initials thereby responding by new root system capable of replacing the original one when it has been damaged by submergence [77]. Moreover, Salicylic acid (SA) participates in the waterlogging-tolerance of plants [79]. SA content in waterlogging-tolerant soybean lines increased significantly after waterlogging for 5 or 10 days compared to non-waterlogging conditions while SA content in sensitive lines exhibited no significant change, implying that SA mediates waterlogging-tolerance of soybean through regulating the formation of aerenchyma or adventitious roots. One gene, *Glyma.08 g348500* possesses SA responsive element (TCA-element). These warrant functional validation of the eight candidate and three hub genes to unravel their actual regulatory mechanism in seed flooding tolerance.

## Conclusions

In this study, two distinct models of GWAS were used to decipher the genetic architecture underlying seed flooding stress tolerance in soybean. The major SNPs, Gm_08_11971416, Gm_08_46239716, Gm_05_1000479, and Gm_01_53535790 were identified to be associated with seed flooding tolerance related traits, viz., EC, GR, ShL and RL. Based on GO enrichment analysis, gene functional annotations, and PPI network analysis, we predicted eight candidate genes and three hub genes with functions directly or indirectly connected to stress defense mechanisms. However, further genetic and molecular analyses are required to validate the functional importance of the putative candidate genes in adaptation to seed flooding stress in soybean. Taken together, these findings provide valuable insight on the genetic basis of soybean seed flooding tolerance, and they could assist MAS in determining the molecular mechanism of seed flooding tolerance in soybean.

## Materials and methods

### Plant materials

A panel of 243 PIs originated from 22 different countries globally was selected from the United States Department of Agriculture, Soybean Germplasm Collection. The seeds of the selected PIs were obtained from the National Center for Soybean Improvement, Nanjing Agricultural University, Nanjing, China. These accessions were planted in 2018 and 2019 at Jiangpu Experimental Station (latitude 32.12°N; longitude 118.37°E) of Nanjing Agricultural University in Nanjing, Jiangsu Province of China. All the accessions were grown in a randomized complete block design with three replications every year. Additional File 6: Table S8 gives details information about the 243 soybean PIs accessions used in this study.

### Phenotypic evaluation for seed-flooding tolerance

Soybean PIs accessions were phenotypically evaluated for seed flooding stress following a previously described method [12]. Briefly, good quality and healthy seeds were surface sterilized by soaking in 70% ethanol for 10 s to remove the contaminants. These seeds were then rinsed in distilled water three times. Twenty seeds of each accession were dipped into 350 ml plastic cups containing 50 ml distilled water covered with sterilized petri dishes for 72 h of seed flooding stress and incubated in a germination cabinet at 25 °C. The experiment was conducted with two replications arranged in a completely randomized design. All accessions were phenotypically evaluated for four traits viz., EC, GR, ShL, and RL at the germination stage (Additional File 7: Table S9). Immediately after the treatment, a conductivity meter (model: DDS-307A) was utilized to record the EC of steep-water. Germination experiment was carried out using a paper roll method in which seeds were grown for 5 days under normal conditions. The germination of seeds with a radicle length of more than 1 cm was considered. For the control, seeds without seed flooding treatment were grown under the same conditions. Relative values of each trait were obtained by dividing the treatment of each accession by its control.

### Phenotypic data analysis

Analysis of variance (ANOVA) of the phenotypic data was performed using the PROC GLM procedure in SAS 9.4 (SAS Institute, Inc., Cary, NC, United States). Broad-sense heritability (*h*^2^) of each trait was estimated for the combined environments as $h^2=\sigma_g^2/(\sigma_g^2+\sigma_{ge}^2/n+\sigma_e^2/nr)$ for combined environments, where ${\sigma}_g^2$ represents the genotypic variance, ${\sigma}_{ge}^2$ is the variance of the genotype-by-environment interaction, ${\sigma}_e^2$ is the error variance, n is the number of environments, and r represents the number of replications within each environment [80]. Pearson correlations were also calculated to measure the degree of relationship between each pair of traits, and the individual hypothesis tests of the correlations were performed at *α* = 0.01 using OriginPro 9.0 software (Origin Lab, Corporation, Northampton, USA).

### SNP genotyping and quality control

SNP data of the selected PIs is available at Illumina Infinium SoySNP50K BeadChip database on SoyBase (https://soybase.org/snps/index.php). The details on genotyping and SNP calling procedures for the 42,449 SNPs as described by Song et al. (2013). After removing SNPs with minor allele frequency (MAF) < 0.05, a total of 34,718 SNPs were used for further analysis in this study.

### Population structure and linkage disequilibrium analyses

A neighbor-joining tree was constructed together with Principal component analysis (PCA) was calculated using TASSEL 5.0 software [38]. The 34,718 SNPs were also used to calculate kinship matrixes by the identity-by-state (IBS) method implemented in TASSEL 5.0 to infer population stratification and relatedness among individuals. A heatmap of the kinship matrix of the 243 accessions was constructed with the *kinship 2* package in R (R Core Team, 2019).

Pairwise linkage disequilibrium (LD) between 34,718 SNPs with a missing rate < 10% and MAF ≥ 0.05 was estimated using squared allele frequency correlations (*r*^*2*^) using the RTM-GWAS V1.1 software [81]. The panel’s LD decay rate was calculated as the chromosomal distance when *r*^*2*^ fell to half its highest value. The average LD decay figure was drawn by GraphPad Prism version 5.01 (GraphPad Software, San Diego California USA, www.graphpad.com) using *r*^*2*^ for SNPs with pairwise distances less than 5 Mb in each chromosome.

### Genome-wide association study and haplotype block analysis

An association analysis was performed for four traits viz., EC, GR, ShL, and RL through two distinct models, Mixed Linear Model (MLM) and Multi-Locus Random-SNP-Effect Mixed Linear Model (mrMLM) for each year plus average of phenotypic data across the 2 years. The MLM was run with the TASSEL 5.0 software, and the K matrix serves as a random effect in this model. However, mrMLM was carried out using the R package *mrMLM.GUI* version 2.1 [82]. The significance threshold used in the present study was set at -log_10_ (1/m) where m = the number of markers, thus -log_10_(1/34,718) = 4.54 as the Bonferroni correction line, however this threshold was adjusted to *-log*_*10*_ *(P)* ≥ 3.5 and logarithm of odd (LOD) = 3 were used to declare SNP-trait association in MLM [41, 42] and mrMLM [46] respectively.

Haplotype block analysis of the stable SNPs was investigated with Haploview software version 4.2 with the four-gamete rule method [83, 84]. Duncan Range Multiple test (pairwise comparison) was used to evaluate variation in seed flooding tolerance among accession groupings in each haplotype block at the significant level of *P* ≤ 0.05.

### Candidate and hub genes identification

The stable SNPs (detected for at least 2 traits by either of the models or both) were used to mine putative candidate genes at 500 kb up- and down-stream of the SNP position by using Glyma2.0 models on SoyBase (http:www.soybase.org). The model genes were retrieved from each of the regions for candidate gene analysis. Gene ontology (GO) enrichment analysis was conducted for all the model genes within the four SNPs (±500 kb) using agriGO (http://bioinfo.cau.edu.cn/agriGO) [85], in which the parameters of singular enrichment analysis (SEA) tool following the default settings and *G. max* gene model as a reference background. Candidate genes associated with seed flooding tolerance were predicted based on GO enrichment analysis and gene functional annotations from the Phytozome (https://phytozome.jgi.doe.gov) and SoyBase (http://www.soybase.org) databases. These model genes were also used to construct probable protein-protein interaction (PPI) network with a publicly available online database Search Tool for Retrieval of Interacting Genes/Proteins (STRING) [86]. Credible PPI interactions were further visualized with the network analyst 3.0 to identify hub genes [87]. The expression data of predicted candidate genes were obtained from transcriptome profile data publicly available on SoyBase (https://soybase.org/soyseq/) developed by Severin et al. [75] and heatmapped by TBTool [88]. In addition, the promoter regions of predicted genes that may be involved in seed-flooding tolerance were further analyzed following the procedure outlined by with Karikari et al. [23]

## Supplementary Information

**Additional file 1: Table S1.** SNPs markers associated with electrical conductivity (EC), germination rate (GR), Shoot Length (ShL), and Root Length (RL) via MLM.

**Additional file 2: Table S2.** SNPs markers associated with electrical conductivity (EC), germination rate (GR), Shoot Length (ShL), and Root Length (RL) via mrMLM.

**Additional file 3: Table S3.** One hundred fifteen (115) model genes were found within the region of Gm_08_11,971,416 SNP using Glyma2.0 models in SoyBase. **Table S4.** One hundred thirty (130) model genes were found within the region of Gm_08_46,239,716 SNP using Glyma2.0 models in SoyBase. **Table S5.** One hundred thirteen (113) model genes were found within the region of Gm_05_1,000,479 SNP using Glyma2.0 models in SoyBase. **Table S6.** One hundred twenty-five (125) model genes were found within the region of Gm_01_53,535,790 SNP using Glyma2.0 models in SoyBase.

**Additional file 4: Figure S1.** Heat map exhibiting the expression profiles of candidate genes among the different soybean tissues and development stages.

**Additional file 5: Table S7.** Cis-acting regulating elements related to seed flooding tolerance obtained from PlantCare database.

**Additional file 6: Table S8.** A core set of 243 soybean plant introductions (PIs) selected from the USDA Soybean Germplasm Collection.

**Additional file 7: Table S9.** Phenotypic data (average) of 243 PI accessions used in this study.

## Data Availability

With the exception of SNP datasets, all data generated or analyzed during this study are included in this article and its supplementary information files. The SNP dataset used in the current study were selected from United States Department of Agriculture, Soybean Germplasm Collection sequenced by Illumina Infinium SoySNP50K BeadChip Technology available on SoyBase (https://soybase.org/snps/index.php). Phenotypic data generated for the 2 years (2018 and 2019) and combined years (CE) have been included in the supplementary Tables (Additional File 5: Supplementary Table 8).

## Abbreviations

GWAS: Genome-wide association study
MLM: Mixed Linear Model
mrMLM: Multi-Locus Random-SNP-Effect MLM
EC: Electrical conductivity
GR: Germination rate
ShL: Shoot length
RL: Root length
GO: Gene ontology
PPI: Protein-protein interaction
QTL: Quantitative trait locus
QTNs: Quantitative trait nucleotides
