# Supplementary material for: Genome-wide association study uncovers major genetic loci associated with seed flooding tolerance in soybean
Source: BMC Plant Biol. 2021 Oct 29;21:497. doi: 10.1186/s12870-021-03268-z (PMC8555181; doi:10.1186/s12870-021-03268-z)
Supplement: Supplementary file 4 — Additional file 4: Figure S1. Heat map exhibiting the expression profiles of candidate genes among the different soybean tissues and development stages. [file 12870_2021_3268_MOESM4_ESM.docx]

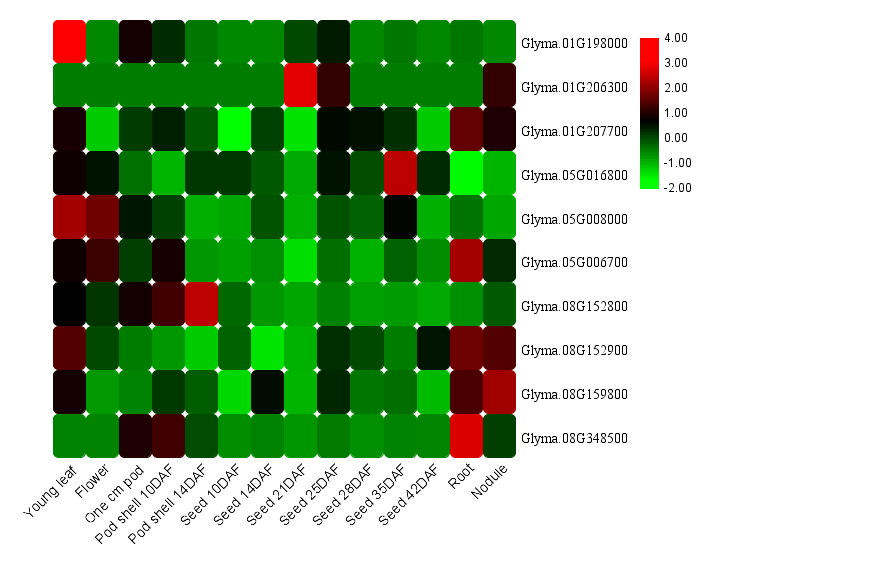


**Fig. S1**. Heat map exhibiting the expression profiles of candidate genes among the different soybean tissues and development stages. Heat map was generated using the RNA-sequencing data downloaded from online database SoyBase (<https://soybase.org/soyseq/>). DAF=Days after flowering.
